# Ophthalmic complications following maxillofacial and midface fractures: a systematic review”

**DOI:** 10.1186/s40902-025-00498-1

**Published:** 2026-04-23

**Authors:** Rawan Affat Alanazi, Abdulaziz Almutairi, Sarah Almuwarraee, Sarah Amuatrafi, Shaden Bahatheq, Ahmed Saad Al zomia, Raghad Alotaibi, Rudaynah Haddad, Mashael Bajunaid, Rund Almohaish, Jana Batarji, Zainab Alasfour, Shaikha Aleid

**Affiliations:** 1 Arabian Gulf University, Manama, Bahrain; 2 King Abdulaziz University, Jeddah, Saudi Arabia; 3 Taibah University, Medina, Saudi Arabia; 4 King Saud bin Abdulaziz University for Health Sciences, Jeddah, Saudi Arabia; 5 King Khalid University, Abhā, Saudi Arabia; 6 Alrayan collage, Medinah, Saudi Arabia; 7 Taif University, Ta’if, Saudi Arabia; 8 King Faisal University, Al Hufūf, Saudi Arabia; 9 King Fahd Hospital of the University, Khobar, Saudi Arabia

**Keywords:** Maxillofacial fractures, Ocular injuries, Systematic review, Ophthalmic complications

## Abstract

Maxillofacial fractures can lead to significant ophthalmic complications, affecting patient outcomes. This systematic review synthesizes current evidence regarding the incidence, causes, and types of ocular injuries associated with facial fractures, while evaluating inter-study variability and risk of bias. Following the PRISMA guidelines, a comprehensive search of PubMed, Cochrane, and Web of Science databases was performed. Twenty-one studies, including 7,998 participants, met the inclusion criteria. Among them, 3,693 patients presented with ophthalmic complications after facial fractures. Orbital fractures were the most prevalent, with injuries primarily resulting from road traffic accidents and assaults. Subconjunctival hemorrhage was the most common ocular injury. Heterogeneity across studies was assessed descriptively due to the diversity of study designs and outcomes, while risk of bias was evaluated using adapted criteria for observational studies. The findings underscore the necessity for standardized diagnostic and reporting criteria to enable future quantitative synthesis and guide clinical protocols for early detection and management of ocular complications.

## Introduction

Despite comprising only 0.3% of the body’s surface area, the human eye, particularly in the context of midface injuries, is vulnerable to harm despite being protected by surrounding fat and bone [1]. Several factors, including the firm periorbital structures, the unique design of the globe, the optic nerve’s robust bony protection as it enters the orbit, aerated paranasal sinuses, and the patient’s self-preservation mechanisms, collectively safeguard the globe, making vision loss after facial trauma uncommon [2]. Unfortunately, these protective mechanisms are often inadequate in preventing eye injuries in cases of craniofacial trauma [3]. Moreover, ocular injuries resulting from head trauma have become a significant concern in the context of potential blindness [4].

Facial fractures are frequently encountered in emergency rooms and pose significant public health challenges due to their associated costs, as well as functional and aesthetic issues patients may experience [5]. Globally, maxillofacial skeletal fractures constitute as a significant portion of annual trauma cases [6], with approximately 200–300 per 100,000 individuals requiring hospitalization due to head injury, and around 25% of these cases presenting with ocular and visual defects [7]. Among facial fractures, the mandible is the most commonly affected bone, followed by zygomaticomaxillary complex (ZMC), orbital fractures, naso-orbito-ethmoid (NOE) fractures, and Le Fort fractures. Midface injuries, such as ZMC fractures, orbital fractures, Le Fort II, and Le Fort III fractures, have a significantly higher risk of vision impairment compared to other facial bone fractures [8].

Ocular trauma is a significant global concern, responsible for causing blindness in over half a million individuals worldwide and resulting in partial loss of sight for many more. It often stands as the primary reason for unilateral vision loss, particularly in developing nations [9]. The incidence of visual sequelae in patients with midface fractures ranges from 2.7% to 9.6%. Literature studies have demonstrated a close association between orbito-zygomatic fractures and ocular complications, indicating a high risk of visual acuity loss and significant morbidity in midface injuries [3, 10, 11]. Following trauma, most patients experienced minor ocular injuries, including subconjunctival hemorrhage, iris sphincter tear, and corneal abrasion. Major injuries, such as ruptured globe and retinal hemorrhage, were observed in 10% of patients. Orbital findings, such as restriction of extraocular movement, were present in 15% of cases. Symptomatic diplopia was noted in 16% of the patients, while traumatic optic neuropathy was a rare occurrence, seen in only a small portion of patients. The etiology of maxillofacial trauma includes road traffic accidents (RTA) (64.0%), assaults (21.0%), falls (5.0%), and other causes [12, 13]. Industrial accidents have also been reported as a cause of trauma following RTA in another study [14]. In developing nations like India, RTAs are a major cause of trauma, while many non-Indian studies point to assaults as the primary etiology for maxillofacial trauma [13].

Male gender shows a significant predilection for trauma, accounting for 85% of cases globally [14]. The long and short posterior ciliary arteries are situated within the muscle cone and enter the eye near the optic nerve. A minor hemorrhage within the cone can exert pressure on these vessels, resulting in ischemic neuropathy and subsequent vision loss. Additional mechanisms of injury encompass retinal edema or detachment, blockage of retinal vessels, and indirect damage to the cerebral cortex or optic chiasm [15, 16].

Oral and maxillofacial surgeons are often the first clinicians to encounter ophthalmic injuries associated with facial trauma. Thus, they play a crucial role in performing the initial ophthalmic examination and treatment. Prompt diagnosis of potentially serious ocular injuries is vital to mitigate long-term effects and for legal reasons. Prioritizing the treatment of eye problems is essential, as fixing fractures before addressing ocular injuries may further harm visual outcomes. A thorough understanding of ophthalmic complications related to facial fractures is paramount to ensure the success of treatment by oral and maxillofacial surgeons [17]. Magarakis et al. [3], conducted a comprehensive systematic review of the literature to investigate the incidence of ocular injury, visual impairment, and blindness in patients with facial fractures. The primary aim was to identify any potential associations between these ocular injuries and specific fracture patterns. However, it was observed that the available studies presented inconsistent data, which may limit the ability to draw definitive conclusions. Therefore, the primary objective of this systematic review is to identify fracture–injury patterns that predict vision-threatening complications in patients with maxillofacial trauma, based on a synthesis of the available literature.Unlike previous systematic reviews (e.g., Magarakis et al.,2012), the present study incorporates recent data up to 2023 and includes a broader international sample. It also uniquelycategorizes fracture types, mechanisms of trauma, andophthalmic outcomes, providing updated clinical insight intopatterns of ocular complications in maxillofacial trauma.

## Materials and methods

### Literature search strategy

This systematic review was prospectively registered in PROSPERO (CRD42023443936) and conducted in adherence to the Preferred Reporting Items for Systematic Reviews and Meta-Analyses (PRISMA) guidelines. A comprehensive electronic search was conducted of PubMed, Cochrane, and Web of Science databases for studies published between 1991 and June 2023. The search strategy was designed independently by one of the authors and approved by the rest of the study team. An amalgamation of Medical Subject Heading (MeSH), such as ("facial fracture" or "maxillofacial fracture" or "orbital fracture”) and ("ophthalmic complications" or "ocular injuries") were used to inclusively identify all studies pertaining to ocular complications associated with facial fractures patients. References of the selected studies were further reviewed to identify missing articles. The full search strategy can be found in the supplemental information. Additionally, Due to substantial heterogeneity across studies in design, reporting, and outcome measures, a meta-analysis was deemed inappropriate. Therefore, a descriptive synthesis of findings was performed in accordance with PRISMA guidance.

### Assessment of homogeneity

Due to the diversity in study design, populations, and outcome measures, statistical pooling was not feasible. Instead, heterogeneity was assessed descriptively, focusing on variations in patient demographics, fracture classification, and outcome reporting. Differences among studies were documented and discussed narratively, and sources of inconsistency were identified to highlight methodological limitations and guide recommendations for future systematic reviews.

### Inclusion criteria

- Clinical studies (retrospective or prospective) reporting on pediatric or adult patients with maxillofacial fractures.
- Studies that reported one or more of the following ophthalmic complications: eyelid laceration, corneal abrasion, ruptured globe, intraocular hemorrhage, lens dislocation, or retinal detachment.
- Studies published in English.

### Exclusion criteria

- Studies not related to ophthalmic complications.
- Non-English publications.
- Studies with fewer than 10 patients.
- Studies that did not report outcomes of interest.
- Case reports, reviews, or non-clinical studies.

### Selection of articles and data extraction

All selected records from the primary search were imported into Rayyan for duplicate checking. After deduplication, the results were screened by five authors for relevance based on title and abstract. The full texts of the selected studies were then independently reviewed by another five authors for final inclusion or exclusion. Any disagreements during the screening process were resolved by a single author.

Data extracted from the included studies encompassed the year of publication, country or region, study design, sample size, mean age at presentation, most common fracture type, complications, and mechanism of trauma. For data analysis, the studies were categorized based on common causes of fracture, types of injury, and mechanisms of trauma.

### Risk of bias assessment

The risk of bias in the included studies was assessed using the Joanna Briggs Institute (JBI) checklist for prevalence studies. Two reviewers independently evaluated each study across domains including sampling, measurement, and statistical analysis. Discrepancies were resolved through discussion or by a third reviewer.

## Results

**Fig. 1:**
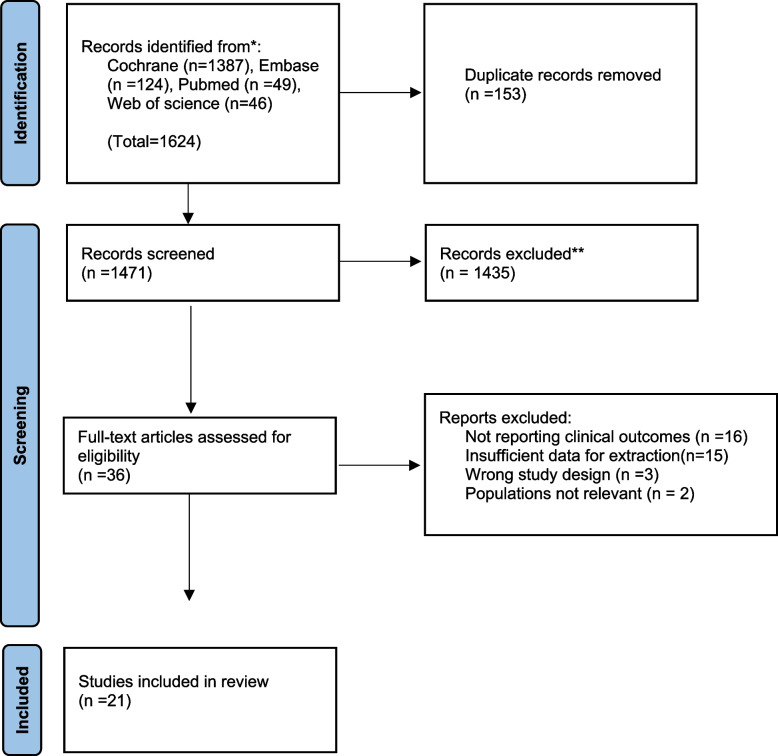
The studies were finalized after a comprehensive literature search strategy and consideration of inclusion and exclusion criteria

The systematic review included 21 studies conducted between 1991 and 2022 (Fig. 1). The number of participants in the included studies was 7,998, with 3,693 patients experiencing ophthalmic complications. Regarding the study designs, the majority of studies (16 studies) followed a retrospective approach; three studies utilized a prospective design, one was a cross-sectional study, and another employed a decennary observational study design. Most studies were conducted in the United States (*n* = 6), followed by China (*n* = 3), Korea (*n* = 3), Australia (*n* = 2), Canada (*n* = 1), Pakistan (*n* = 1), Iran (*n* = 1), and Burkina Faso (*n* = 1). Moreover, the studies encompassed a wide age range from 4 months to 91 years of age. Male patients consisted of the highest proportion in most studies. All details are described (Table 1).

**Table 1:** Total characteristics of the included papers. – means not available

No	Authors, year of publication	Country	Study design	Sample size		Age	Gender distribution		
				Total patients	Patient with ophthalmic complications		Total	Male	Female
1	Al-Qurainy IA et al., 1991 [18]	-	Prospective study	363	37	-	363	-	-
2	Lim LH et al., 1993 [19]	Australia	Prospective study	839	33	Range: 15–88 years	839	678	161
3	Fulcher TP and Sullivan TJ, 2003 [20]	Australia	Retrospective cohort study	21	8	-	21	16	5
4	Nagase DY et al., 2006 [21]	-	Retrospective review	266	100	-	-	-	-
5	Chi MJ et al. 2010 [22]	Korea	Retrospective study	733	733	Mean (SD): 30.7 (13.0) years	733	549	184
						Range: 2–73 years			
6	Shin JW et al., 2013 [23]	Korea	Retrospective study	952	91	Mean (SD): 31.5 (15.82) years	952	773	179
						Range: 2–88 years			
7	Hink EM, et al., 2014 [24]	USA	Retrospective case series	312	192	Range: 4 months-16 years	312	192	120
8	Zhou HH et al., 2014 [8]	China	Retrospective study	881	176	Mean (SD): 32.4 (11.27)	209	176	33
9	Sheng I et al., 2015 [25]	New Jersey’s	Retrospective chart review	90	90	Mean (SD): 80.01 (8.23) years	90	36	54
						Range:65–96 years			
10	Malik TG et al., 2015 [26]	Pakistan	Descriptive, retrospective study	42	-	Mean: 38.5 years	42	36	6
						Range: 2–75 years			
11	Su Y et al., 2015 [27]	China	Retrospective study	83	83	Range: 3–18 years	83	59	24
12	HoQ, 2017 [28]	USA	Prospective study	80	72	Average: 22.1 years	80	46	34
						Range: 8–89 years			
13	Chen X et al., 2017 [29]	China	Retrospective Study	99	99	Mean (SD): 33 (19) years	197	175	223
14	Ross M et al., 2017 [30]	Canada	Retrospective study	73	10	Median: 42 years	73	50	23
15	Béogo R et al., 2018 [31]	Burkina Faso	Retrospective study	907	557	Mean: 31.2	907	795	112
						Range: 6–59			
16	Aslan F and Ozen O, 2019 [32]	USA	Retrospective study	286	83	-	83	20	63
17	Terrill SB et al., 2020 [33]	USA	Retrospective study	460	373	Range: 0–95 years	460	398	122
18	Noh H et al., 2021 [34]	Korea	Retrospective study	200	129	Mean (SD): 40.5 (19.9) years	158	120	38
						Range: 6- 88 years			
19	Dalband M et al., 2022 [35]	Iran	Cross-sectional study	174	110	Mean (SD): 31.1 (12.35)	174	151	23
						Range: 7–78 years			
20	Singh I et al., 2022 [36]	-	A decennary descriptive study	686	370	NA	686	566	120
21	Ray CN et al., 2022 [37]	Texas	Retrospective study	451	347	Mean: 30 years	451	355	96
						Range: 1–91 years			

In the various studies conducted over the years, the results regarding the mechanisms and most common causes of trauma to the eye and fracture types were reported (Table 2). The studies reported a wide range of causes of trauma, including road traffic accidents, assaults, falls, sports-related injuries, work-related injuries, blunt trauma, penetrating injuries, and gunshot wounds. Road traffic accidents were the most frequent cause of eye trauma in several studies (1468 cases, 18.4%), with the highest 819 cases reported by Béogo (2018) [31]. The assault was another significant cause (1161 cases, 14.5%), with Shin JW et al. (2013) reporting 387 patients [23], and Chi (2010) documenting 365 cases [22]. Falls were also a common cause (753 cases, 9.4%), with 266 patients in the Shin JW et al. (2013) study [23] and 222 cases in the Terrill (2020) study resulting from falls [33]. Although generally less frequent, sports-related injuries were observed across multiple studies among 405 cases (5.1%), with Hink et al. [24] reporting 114 sports-related cases. Other causes of eye trauma were mentioned in some studies, including work-related injuries and gunshots (411 cases, 5.1%).

**Table 2:** Mechanisms of trauma and fracture types among the patients

No	Authors, year of publication	Mechanism and most common cause of Trauma					Fracture types					
		Road traffic accidents	Assault	Falls	Sports	Other	Orbital	Midfacial	Mandibular	Nasal	Zygomatic	Other
1	Al-Qurainy IA et al., 1991 [18]	-	-	-	-	-	-	-	-	-	-	-
	Lim LH et al., 1993 [19]	33	4	4	1	Work-related injuries (3)	6	-	-	2	11	LeFort. pan facial and other complex fractures (14)
	Fulcher TP and Sullivan TJ, 2003 [20]	11	-	3	-	Blunt trauma (4) and penetrating orbital injuries (3)	21	-	-	-	-	Clavicle fractures (1). Lumber vertebral fracture (1), Femur fracture with hemothorax (1), and rib and thoracolumbar vertebral fractures (1)
	Nagase DY et al., 2006 [21]	-	-	-	-	-	4	2	4	1	4	-
	Chi MJ et al. 2010 [22]	94	365	137	48	29	757	55	-	50	34	Le fort (5)
	Shin JW et al., 2013 [23]	116	387	266	77	Bumping or hitting (106)	983	-	-	-	-	
	Hink EM, et al., 2014 [24]	48	11	86	114	Face blow (3), Gunshot (2)	312	88	15	23	39	Cribriform fractures (32), parietal fractures (23), and occipital fractures (8) Maxillary fractures (53), Le Fort fractures (18) and open fractures (37)
	Zhou HH et al., 2014 [8]	97	30	10	1	Bicycle (6), Motorcycle (43) and work-related (1)	-	131	13	-	-	Midfacial and Mandible (132), and Multimandible (378)
9	Sheng I et al., 2015 [25]	-	-	-	-	-	10	-	-	-	-	-
10	Malik TG et al., 2015 [26]	32	-	5	-	Face blow (3), and gunshot (2)	16	-	-	-	-	Multiple unspecified fractures (15)
11	Su Y et al., 2015 [27]	22	10	12	12	Unknown causes (2)	37	-	-	-	-	Trapdoor fracture (13) and Medial-floor (17)
12	Ho Q, 2017 [28]	12	31	27	7	Others (3)	80	-	-	-	-	-
13	Chen X et al., 2017 [29]	17	17	-	42	Gunshot (5) Explosive (42) and work-related (46)	98	-	-	-	-	-
14	Ross M et al., 2017 [30]	5	29	25	3	Bike accidents (7), accidental trauma with an elbow to the face (2), and all-terrain-vehicle accidents (2)	73	-	-	-	-	-
15	Béogo R et al., 2018 [31]	819	-	-	-	-	-	-	-	-	353	Nasofronto-orbito-ethmoidal (NFOE) (25), Le fort (125)
16	Aslan F and Ozen O, 2019 [32]	23	19	21	1	Blunt trauma (12), and unknown causes (7)	65	37	-	-	42	-
17	Terrill SB et al., 2020 [33]	117	222	112	-	gunshot wound (9)	567	-	-	-	-	-
18	Noh H et al., 2021 [34]	22	36	45	32	23	158	-	-	-	23	-
19	Dalband M et al., 2022 [35]	-	-	-	-	-	98	-	90	20	89	Frontal (12) and Lefort (29)
20	Singh I et al., 2022 [36]	-	-	-	-	-	351	105	-	-	249	-
21	Ray CN et al., 2022 [37]	-	-	-	67	Work-related injury 34. Other undefined causes 12	451	-	-	-	-	-
**Total number of patients**		1468	1161	753	405	411	4087	418	122	96	844	930
**Percantage**		18.4	14.5	9.4	5.1	5.1	51.1	5.2	1.5	1.2	10.6	11.6

Moreover, several studies were examined in a comprehensive analysis of fractures. Orbital fractures were reported in most studies (4087 cases, 51.1%), with 983 cases reported in a study by Shin et al. [23], and 757 and 567 in studies by Chi et al. (2010) [22] and Terrill SB et al. (2020) [33], respectively. Other fracture types documented in studies were midfacial (418 cases, 5.2%), mandibular (122 cases, 1.5%), nasal (96 cases, 1.2%), zygomatic (844 cases, 10.6%), and other fractures (930 cases, 11.6%). All details are provided (Table 2).

A comprehensive overview of the types of ocular injuries reported in various studies is presented (Table 3). The reported ocular injuries included eyelid injuries (ecchymosis, swelling, laceration, hyperemia, ptosis), eyeball injuries (ruptured globe), conjunctival and subconjunctival injuries (hemorrhage, hyperemia, swelling), scleral injuries (hyperemia, laceration), periorbital injuries (ecchymosis, swelling, laceration), lens injuries (dislocation, traumatic cataract), and other ocular injuries. The most frequent ocular injury types reported in the included studies were subconjunctival injuries (hemorrhage, hyperemia, swelling) which affected 1148 cases (31.1%), followed by periorbital injuries (ecchymosis, swelling, laceration) (890 cases, 24.1%), and eyelid injuries (821 cases, 22.2%). On the other hand, the least frequent ocular injury type diagnosed was lens injury (dislocation, traumatic cataract), which was reported among only six patients (0.2%).

**Table 3:** Types of ocular injuries in the included studies

Authors, year of publication	Eyelid (ecchymosis, swelling, laceration, hyperemia,Ptosis)	Eyeball injury (Ruptured globe)	Conjunctival (hemorrhage, hyperemia, Swelling)	Subconjunctival (hemorrhage, hyperemia, Swelling)	Scleral (hyperemia, laceration)	Periorbital (ecchymosis, swelling, laceration)	Blindness	Diplopia	Corneal/Corneoscleral (abrasion, laceration)	AC (hyphema)	Lens (dislocation, traumatic cataract)	Pupil abnormality	Injuries of external muscle	Vitreous (hemorrhage)	Retinal (tear, edema, hemorrhage, Macular hemorrhage, commoti retina)	choroidal hemorrhage/rupture	Optic nerve damage	Poor vision	Visual field loss	Lacrimal apparatus injury	Enophthalmous	Enucleation/Exenteration
Al-Qurainy IA et al., 1991 [18]	Eyelid laceration (6)	-	-	-	-	-	-	3	-	-	1	3	-	1	2	-	-	-	-	-	4	-
Lim LH et al., 1993 [19]	-	-	1	-	-	-	-	-	4	-	-	-	-	3	13	1	5	-	-	-	-	3
Fulcher TP and Sullivan TJ, 2003 [20]	Eyelid laceration 1	2	-	-	-	-	1	4	-	-	-	-	4	-	-	-	3	-	-	-	-	-
Nagase DY et al., 2006 [21]	2	4	-	-	-	1	9	8	-	-	-	-	-	-	6	1	4	10	-	-	-	-
Chi MJ et al. 2010 [22]	729[672,0,48,0,9]	4	-	482(482,0,0)	-	672(672, 0,0)	-	325	160	130	2	-	436	8	51(4,0,5,0,42)	-	13	-	-	8	131	-
Shin JW et al., 2013 [23]	-	-	-	-	-	-	1	263	81	76	1	-	123	-	2(0,0,1,0,1)	-	2	-	-	-	31	-
Hink EM, et al., 2014 [24]	-	-	-	-	-	-	-	8	-	-	2		16	1	17	-	9	9	-	5	6	-
Zhou HH et al., 2014 [8]	49 (15,21,8,1,4)	1	63 (0,62,1)	-	7	137 (108,18,11)	1	5	-	-	-	-	12	-	-	-	-	3	5	1	4	4
Sheng I et al., 2015 [25]	-	-	46		26	-	-	-	40	-	-	38	-	46	24	34	-	-	-	-	-	4
Malik TG et al., 2015 [26, 27]	-	3	22	-	-	-	-	-	2	2	-	-	9	3	2	-	11	-	1	-	9	-
Su Y et al., 2015 [27]	-	-	-	-	-	-	-	71	-	-	-	-	116	-	-	-	-	-	-	-	39	-
Ho Q, 2017 [28]	-	1	-	34 (34,0,0)	scleral tear 1	-	2	15	2	2	-	5	-	-	-	-	-	10	-	-	-	-
Chen X et al., 2017 [29]	-	-	-	-	-	-	-	-	-	-	-	-	-	-	-	-	-	-	62	-	0	-
Ross M et al., 2017 [30]	1 (0,0,1,0,0)	1	-	40 (36,0,4)	-	39(18,21)	-	-	2	-	-	1	1	-	3 (0,0,2,0,1)	-	1	-	-	-	-	1
Béogo R et al., 2018 [31]	-	18	-	-	-	-	10	-	-	-	-	-	-	3	10	-	13	-	-	-	-	-
Aslan F and Ozen O, 2019 [32]	3	-	-	-	-	-	-	-	1	-	-	2	9	-	119	-	9	-	-	-	-	1
Terrill SB et al., 2020 [33]	-	13	13	246 (246,0,0)	-	-	-	1	42	23	-	82	-	-	80 (1,0,11,4,64)	5	8	63	-	-	1	-
Noh H et al., 2021 [34]	-	5	-	-	-	-	-	-	-	-	-	-	-	-	-	-	-	-	-	-	-	-
Dalband M et al., 2022 [35, 36]	-	7	-	74	14	-	-	-	-	-	-	-	-	-	-	-	19	15	-	-	-	13
Singh I et al., 2022 [36]	-	-	-	219	-	-	4	-	15		-	-	-	-	4	-	-	-	-	-	-	-
Ray CN et al., 2022 [37]	30	-	-	53	-	41	-	97	31	-	-	14	-	5	120	9	3	445	-	-	-	2
Total number of patients	821	59	145	1148	48	890	28	800	380	248	6	145	726	70	453	50	100	555	68	14	225	28
Percentage of ocular injury	22.2	1.6	3.9	31.1	1.3	24.1	0.8	21.7	10.3	6.7	0.2	3.9	19.7	1.9	12.3	1.4	2.7	15.0	1.8	0.4	6.1	0.8

## Discussion

This systematic review examined the range of ophthalmic complications associated with maxillofacial fractures and explored whether these complications correspond to specific patterns of facial injury. Across 21 studies involving 7,998 patients, ophthalmic complications were reported in approximately 46% of cases, confirming that ocular involvement is a common consequence of facial trauma. Orbital fractures were consistently the most frequent fracture type, and the majority of injuries resulted from road traffic accidents and assaults [22, 36, 38, 39]. Subconjunctival hemorrhage was the most commonly reported ocular finding.

While previous systematic reviews have addressed ocular trauma in the context of facial fractures, the present review provides an updated synthesis of the literature by incorporating recent studies up to 2023 and including a broader international sample. It also offers a more detailed categorization of fracture types, mechanisms of trauma, and associated ophthalmic outcomes. Due to marked heterogeneity among the included studies regarding diagnostic criteria, reporting methods, and length of follow-up, conducting a meta-analysis was not feasible. Consequently, this review presents a structured descriptive analysis that clarifies patterns and clinical implications while highlighting existing gaps in evidence (Table 4).

**Table 4:** Risk of Bias Assessment of Included Studies Using the JBI Checklist for Prevalence Studies

Study (Author, Year)	1. Sample Frame	2. Sampling Method	3. Sample Size	4. Subjects & Setting	5. Data Analysis	6. Coverage of Identified Sample	7. Objective Measurement	8. Statistical Analysis	Overall Risk of Bias
Al-Qurainy IA et al., 1991 [18]	**U**	**L**	**L**	**L**	**L**	**L**	**L**	**L**	**Low**
Lim LH et al., 1993 [19]	**L**	**L**	**L**	**L**	**L**	**L**	**L**	**L**	**Low**
Fulcher TP and Sullivan TJ, 2003 [20]	**U**	**L**	**H**	**L**	**L**	**U**	**L**	**L**	**Moderate**
Nagase DY et al., 2006 [21]	**U**	**L**	**L**	**L**	**L**	**U**	**L**	**L**	**Low**
Chi MJ et al., 2010 [22]	**L**	**L**	**L**	**L**	**L**	**L**	**L**	**L**	**Low**
Shin JW et al., 2013 [23]	**L**	**L**	**L**	**L**	**L**	**U**	**L**	**L**	**Low**
Zhou HH et al., 2014 [8]	**L**	**L**	**L**	**L**	**L**	**L**	**L**	**L**	**Low**
Hink EM et al., 2014 [24]	**U**	**L**	**L**	**L**	**L**	**U**	**L**	**L**	**Low**
Malik TG et al., 2015 [26]	**U**	**L**	**H**	**L**	**L**	**U**	**L**	**L**	**Moderate**
Su Y et al., 2015 [27]	**U**	**L**	**L**	**L**	**L**	**U**	**L**	**L**	**Low**
Sheng I et al., 2015 [25]	**U**	**L**	**L**	**L**	**L**	**U**	**L**	**L**	**Low**
Ho TQ et al., 2017 [28]	**L**	**L**	**L**	**L**	**L**	**L**	**L**	**L**	**Low**
Chen X et al., 2017 [29]	**U**	**L**	**L**	**L**	**L**	**U**	**L**	**L**	**Low**
Ross M et al., 2017 [30]	**L**	**L**	**H**	**L**	**L**	**L**	**L**	**L**	**Low**
Béogo R et al., 2018 [31]	**L**	**L**	**L**	**L**	**L**	**U**	**L**	**L**	**Low**
Aslan F and Ozen O, 2019 [32]	**L**	**L**	**L**	**L**	**L**	**L**	**L**	**L**	**Low**
Terrill SB et al., 2020 [33]	**L**	**L**	**L**	**L**	**L**	**L**	**L**	**L**	**Low**
Noh H et al., 2021 [34]	**L**	**L**	**L**	**L**	**L**	**L**	**L**	**L**	**Low**
Dalband M et al., 2022 [35]	**U**	**L**	**L**	**L**	**L**	**U**	**L**	**L**	**Low**
Singh I et al., 2022 [36]	**L**	**L**	**L**	**L**	**L**	**U**	**L**	**L**	**Low**
Ray CN et al., 2022 [37]	**L**	**L**	**L**	**L**	**L**	**U**	**L**	**L**	**Low**

*Abbreviations: L* Low risk of bias, *H* High risk of bias, *U* Unclear risk of bias, *N/A* Not applicable

Male patients accounted for the majority of trauma cases (78.7%), consistent with global data reflecting greater exposure to high-risk activities among males [3]. However, no significant association was identified between sex and the likelihood of developing ophthalmic complications once a maxillofacial fracture had occurred [3]. Most patients sustained minor ocular injuries such as subconjunctival hemorrhage, corneal abrasions, or iris sphincter tears. Severe injuries, including ruptured globe, retinal hemorrhage, or optic nerve damage, were less common but clinically significant, occurring in approximately 10% of reported cases [10]. These findings underscore the importance of prompt ophthalmologic evaluation in all patients with facial fractures, particularly midfacial and orbital injuries, as delayed detection of ocular damage remains a major cause of preventable visual loss [22, 33, 40, 41].

Subconjunctival hemorrhage (SCH) was the most frequently documented ocular finding and may present either with or without a visible posterior limit. SCH with a posterior limit is typically confined to the anterior bulbar conjunctiva and usually results from rupture of superficial conjunctival vessels secondary to blunt trauma or increased venous pressure. This form is generally benign and self-limiting. In contrast, SCH without a posterior limit may extend posteriorly beyond the visible conjunctiva and can indicate deeper orbital pathology. In the setting of maxillofacial trauma, this presentation may reflect retrobulbar hemorrhage, orbital compartment syndrome, posterior orbital tissue disruption, or an associated orbital wall fracture. These conditions may threaten vision and require urgent ophthalmologic assessment and imaging. Therefore, distinguishing between the two presentations of subconjunctival hemorrhage is an important component of early evaluation in facial trauma.

The high frequency of ocular involvement demonstrated in this review supports the need for routine and early ophthalmologic assessment in patients with facial fractures. Multidisciplinary collaboration between maxillofacial surgeons and ophthalmologists is essential to ensure early identification of potentially vision-threatening injuries. Understanding fracture-specific risk patterns may further assist clinicians in prioritizing patients who require more urgent ophthalmic evaluation.

Clinical Implications and Referral Guidance.

Based on the findings of this review, the following fracture patterns and mechanisms should prompt mandatory ophthalmologic consultation: orbital floor and medial wall fractures associated with clinical or radiographic evidence of extraocular muscle entrapment or retrobulbar hemorrhage; zygomaticomaxillary complex fractures accompanied by diplopia, enophthalmos, or subconjunctival hemorrhage without a clearly defined posterior margin; Le Fort II and III fractures, irrespective of the presence or absence of initial ocular symptoms; and any midfacial fracture resulting from high-velocity trauma (e.g., road traffic collisions, assaults with blunt instruments). Early referral enables timely identification and management of vision-threatening complications, including traumatic optic neuropathy, retinal detachment, and globe rupture.

This review also highlights several limitations in the existing literature. Substantial variability was observed across studies in terms of design, injury classification, diagnostic definitions, and follow-up duration. Such heterogeneity limits the comparability of findings and prevents the use of quantitative synthesis methods. Future studies should adopt standardized diagnostic criteria for ocular injuries, consistent fracture classification systems, and prospective multicenter designs. In addition, more attention should be directed toward evaluating fracture severity, combined fracture patterns, and long-term visual outcomes. Improved methodological consistency will allow stronger correlations between fracture characteristics and ophthalmic prognosis and may support the development of clearer clinical guidelines.

## Conclusion

Ophthalmic complications frequently accompany maxillofacial fractures, especially orbital and zygomaticomaxillary injuries [3, 36]. Early multidisciplinary evaluation and standardized reporting can enhance prevention and management strategies for vision-threatening trauma.

## Data Availability

No datasets were generated or analysed during the current study.
