# Correction: Ophthalmic complications following maxillofacial and midface fractures: a systematic review”

**DOI:** 10.1186/s40902-026-00517-9

**Published:** 2026-07-15

**Authors:** Rawan Affat Alanazi, Abdulaziz Almutairi, Sarah Almuwarraee, Sarah Amuatrafi, Shaden Bahatheq, Ahmed Saad Al zomia, Raghad Alotaibi, Rudaynah Haddad, Mashael Bajunaid, Rund Almohaish, Jana Batarji, Zainab Alasfour, Shaikha Aleid

**Affiliations:** 1 Arabian Gulf University, Manama, Bahrain; 2 King Abdulaziz University, Jeddah, Saudi Arabia; 3 Taibah University, Medina, Saudi Arabia; 4 King Saud bin Abdulaziz University for Health Sciences, Jeddah, Saudi Arabia; 5 King Khalid University, Abhā, Saudi Arabia; 6 Alrayan collage, Medinah, Saudi Arabia; 7 Taif University, Ta’if, Saudi Arabia; 8 King Faisal University, Al Hufūf, Saudi Arabia; 9 King Fahd Hospital of the University, Khobar, Saudi Arabia

**Correction to: Maxillofacial Plastic and Reconstructive Surgery 48, 7 (2026)**

**https://doi.org/10.1186/s40902-025-00498-1**

Following publication of the original article [1], the authors reported that the given name of the ninth author should be corrected from “Meshael” to “Mashael”.

The correct author name has been provided in this Correction.

The original article [1] has been corrected.
